# Assessing the Relationship Between Leukocyte Telomere Length and Cancer Risk/Mortality in UK Biobank and TCGA Datasets With the Genetic Risk Score and Mendelian Randomization Approaches

**DOI:** 10.3389/fgene.2020.583106

**Published:** 2020-10-23

**Authors:** Yixin Gao, Yongyue Wei, Xiang Zhou, Shuiping Huang, Huashuo Zhao, Ping Zeng

**Affiliations:** ^1^Department of Epidemiology and Biostatistics, School of Public Health, Xuzhou Medical University, Xuzhou, China; ^2^Department of Biostatistics, School of Public Health, Nanjing Medical University, Nanjing, China; ^3^Department of Biostatistics, School of Public Health, University of Michigan, Ann Arbor, MI, United States; ^4^Center for Statistical Genetics, School of Public Health, University of Michigan, Ann Arbor, MI, United States; ^5^Center for Medical Statistics and Data Analysis, School of Public Health, Xuzhou Medical University, Xuzhou, China

**Keywords:** leukocyte telomere length, cancer, genetic risk score, UK Biobank, TCGA, Mendelian randomization

## Abstract

**Background:**

Telomere length is an important indicator of tumor progression and survival for cancer patients. Previous work investigated the associations between genetically predicted telomere length and cancers; however, the types of cancers investigated in those studies were relatively limited or the telomere length-associated genetic variants employed often came from genome-wide association studies (GWASs) with small sample sizes.

**Methods:**

We constructed the genetic risk score (GRS) for leukocyte telomere length based on 17 associated genetic variants available from the largest telomere length GWAS up to 78,592 individuals. Then, a comprehensive analysis was undertaken to evaluate the association between the constructed GRS and the risk or mortality of a wide range of cancers [i.e., 37 cancers in the UK Biobank and 33 cancers in The Cancer Genome Atlas (TCGA)]. We further applied the two-sample Mendelian randomization (MR) to estimate the causal effect of leukocyte telomere length on UK Biobank cancers via summary statistics.

**Results:**

In the UK Biobank dataset, we found that the GRS of leukocyte telomere length was associated with a decreased risk of nine types of cancer (i.e., significant association with multiple myeloma, chronic lymphocytic leukemia, kidney/renal cell cancer, bladder cancer, malignant melanoma, basal cell carcinoma, and prostate cancer and suggestive association with sarcoma/fibrosarcoma and Hodgkin’s lymphoma/Hodgkin’s disease). In addition, we found that the GRS was suggestively associated with an increased risk of leukemia. In the TCGA dataset, we observed suggestive evidence that the GRS was associated with a high death hazard of rectum adenocarcinoma (READ), sarcoma (SARC), and skin cutaneous melanoma (SKCM), while the GRS was associated with a low death hazard of kidney renal papillary cell carcinoma (KIRP). The results of MR further supported the association for leukocyte telomere length on the risk of malignant melanoma, Hodgkin’s lymphoma/Hodgkin’s disease, chronic lymphocytic leukemia and multiple myeloma.

**Conclusion:**

Our study reveals that telomere played diverse roles in different types of cancers. However, further validations in large-scale prospective studies and deeper investigations of the biologic mechanisms are warranted.

## Introduction

Telomere is a special structure with a 6-bp TTAGGG repeat sequence and plays an important role in genomic stability by protecting DNA against damage and fusion 0 (de Lange, 2005). Due to the inability of DNA polymerase to fully extend the 3′ end of DNA strand, the telomere becomes progressively shorter during each round of cell division. The length of telomere is thus a biomarker of cellular and overall biological aging. Once a critically short telomere length is reached, the cell would be triggered to enter senescence, which would ultimately lead to cell growth arrest or apoptosis (Shay and Wright, 2019). In stem and progenitor cells, the length of telomere is maintained by enzyme telomerase (Hackett and Greider, 2002; Shawi and Autexier, 2008). It is shown that enzyme telomerase is activated in almost all human tumors; such an activation can result in the continuous division of cancer cells and is the key component of the tumorigenic phenotype of human cancer cells (Stewart and Weinberg, 2006; O’Sullivan and Karlseder, 2010).

Prior studies have demonstrated that telomere length is associated with a lot of age-related diseases and disorders (e.g., cancers and neurodegenerative disorders) (Zhu et al., 2011) and that a shorter telomere length in tumor tissues is an important indicator of tumor progression and survival for cancer patients (Ma et al., 2011; Xu et al., 2016). However, not all studies reported consistent findings (Supplementary Table S1), partly reflecting the complicated function of telomere on human cancers. The diversity in cancer types, ethnicities, study designs, measurement methods, and selected tissues for telomere length in previous work further complicates the observed association. Given the severe disease burden of cancers worldwide (Siegel et al., 2019), understanding the association between telomere length and cancers can provide valuable insights into the development of cancers and has the potential to improve the prevention and treatment strategies for cancers.

On the other hand, in the past few years, a number of single nucleotide polymorphisms (SNPs) have been identified to be associated with leukocyte telomere length through genome-wide association studies (GWASs) (Levy et al., 2010; Gu et al., 2011; Mangino et al., 2012; Codd et al., 2013; Pooley et al., 2013; Dorajoo et al., 2019). Relying on associated genetic variants, many studies have been undertaken to investigate the association between genetically predicted leukocyte telomere length and cancers. However, the types of cancers investigated in previous studies (Zhang et al., 2015; Li et al., 2020) were relatively limited. In addition, the telomere length-associated SNPs employed in previous studies (Zhang et al., 2015; Rode et al., 2016; Haycock et al., 2017) often came from GWASs with small sample sizes (Levy et al., 2010; Codd et al., 2013).

Recently, a large-scale GWAS of leukocyte telomere length was conducted with the largest sample size to date (up to ∼80,000) (Li et al., 2020), which allows us to choose more appropriate SNPs to study the multilocus genetic profile of leukocyte telomere length *via* the genetic risk score (GRS) approach (Ripatti et al., 2010; Dudbridge et al., 2013; Eusden et al., 2015; Guo et al., 2016; Goldman, 2017; Tosto et al., 2017; Bogdan et al., 2018; De La Vega and Bustamante, 2018; Zeng et al., 2019b). Briefly, GRS is an efficient and powerful genetic method to explore the association between an exposure and complex diseases by integrating multiple genetic variants with weak effects, and it dramatically enhances the predictability of complex diseases through genetic polymorphisms (Belsky et al., 2013; Khera et al., 2018; Duncan et al., 2019; Khera et al., 2019). Moreover, several cancer-relevant cohorts, such as The UK Biobank (Bycroft et al., 2018) and The Cancer Genome Atlas (TCGA) (Hoadley et al., 2018), have collected a variety of cancer-related omics and clinical information, which makes it feasible to systematically investigate a large number of types of cancers.

Based on these valuable data resources, in the present work, we evaluated the association between leukocyte telomere length and 37 cancers from the UK Biobank cohort as well as 33 cancers from the TCGA dataset using the genetic risk score method. We further applied the two-sample Mendelian randomization (MR) (Burgess et al., 2017; Hartwig et al., 2017) to assess the association between leukocyte telomere length and multiple cancers, for which the summary statistics can be available from the UK Biobank cohort. Our study revealed that telomere played cancer-specific roles and that a shorter leukocyte telomere length can either increase or decrease the risk/mortality of cancers. However, further validations in large-scale prospective studies and deeper investigations of the biological mechanism of leukocyte telomere length on various types of cancers are warranted.

## Materials and Methods

### Selection of Instrumental Variables for Leukocyte Telomere Length

We obtained the summary statistics (e.g., effect size and effect allele) of leukocyte telomere length from the ENGAGE consortium as well as the EPIC-CVD and EPIC-InterAct cohorts (Supplementary Table S2; Li et al., 2020), which was the largest GWAS of telomere length (*N* = 78,592) undertaken in the European population to date. In this study, leukocyte telomere length was measured as a continuous variable and the linear additive regression was implemented to investigate the association for each genetic variant (Li et al., 2020). Particularly, in the association analysis, the age of participants was considered as a covariate to remove the influence of biological age. We selected 17 independent index SNPs that were strongly associated with leukocyte telomere length (*p* < 5.00E-8; see Table 1) to construct GRS. Note that, given the fact that the length of telomere would shorten progressively with age, to facilitate the explanation of our results, we made a sign transformation for the effect sizes of these used SNPs so that the relationship under investigation corresponded to a *shorter* leukocyte telomere length.

**TABLE 1:** Independent index single nucleotide polymorphisms (SNPs) associated with leukocyte telomere length in the European population.

SNP	Chr	Position	Gene	A1/A2	EAF	Beta	SE	*p*	PVE	*F*
rs3219104	1	226,562,621	*PARP1*	C/A	0.83	–0.042	0.006	9.60E-11	6.23E-04	49.0
rs55749605	3	101,232,093	*SENP7*	A/C	0.58	0.037	0.007	2.45E-08	3.55E-04	27.9
rs10936600	3	169,514,585	*TERC*	T/A	0.24	0.086	0.006	7.18E-51	2.61E-03	205.4
rs13137667	4	71,774,347	*MOB1B*	C/T	0.96	–0.077	0.014	2.43E-08	3.85E-04	30.2
rs4691895	4	164,048,199	*NAF1*	C/G	0.78	–0.058	0.006	1.58E-21	1.19E-03	93.4
rs7705526	5	1,285,974	*TERT*	A/C	0.33	–0.082	0.006	5.34E-45	2.37E-03	186.8
rs34991172	6	25,480,328	*CARMIL1*	G/T	0.07	0.061	0.011	6.19E-09	3.91E-04	30.8
rs2736176	6	31,587,561	*PRRC2A*	C/G	0.31	–0.035	0.006	3.53E-10	4.33E-04	34.0
rs59294613	7	124,554,267	*POT1*	A/C	0.29	0.041	0.006	1.17E-13	5.94E-04	46.7
rs9419958	10	105,675,946	*OBFC1*	C/T	0.86	0.064	0.007	5.05E-19	1.06E-03	83.6
rs228595	11	108,105,593	*ATM*	A/G	0.42	0.029	0.005	1.43E-08	4.28E-04	33.6
rs2302588	14	73,404,752	*DCAF4*	C/G	0.10	–0.048	0.008	1.68E-08	4.58E-04	36.0
rs3785074	16	69,406,986	*TERF2*	G/A	0.26	–0.035	0.006	4.64E-10	4.33E-04	34.0
rs62053580	16	74,680,074	*RFWD3*	G/A	0.17	0.039	0.007	4.08E-08	3.95E-04	31.0
rs7194734	16	82,199,980	*MPHOSPH6*	T/C	0.78	0.037	0.006	6.94E-10	4.84E-04	38.0
rs8105767	19	22,215,441	*ZNF208*	G/A	0.30	–0.039	0.005	5.42E-13	7.74E-04	60.8
rs75691080	20	62,269,750	*STMN3*	T/C	0.09	0.067	0.009	5.99E-14	7.05E-04	55.4

*Chr, chromosome; A1, effect allele; A2, alternative allele; EAF, frequency of the effect allele; PVE, proportion of variance explained by the SNP [i.e., ${\text{PVE}}_{j}\,\_={\left({\hat{\beta }}_{j}^{\text{X}}\right)}^{2}/\left({\left({\hat{\beta }}_{j}^{\text{X}}\right)}^{2}+\mathrm{var}\,\left({\hat{\beta }}_{j}^{\text{X}}\right)\times N_{j}\right)$, where ${\hat{\beta }}_{j}^{\text{X}}$ and $\text{var}\,\left({\hat{\beta }}_{j}^{\text{X}}\right)$ are the estimated effect size and variance, respectively, for instrument *j* (Shim et al., 2015)]; *F*, *F* statistic [i.e., *F*_*j*_=PVE_*j*_(*N*_*j*_−1−*k*)/(*k*−*k*×PVE_*j*_), where *Nj* is the sample size for instrument *j* (i.e., Nj = 78,592) and *k* is the number of instruments (Burgess et al., 2011; Burgess and Thompson, 2012). Both PVE and *F* statistic are calculated to validate the issue of weak instruments].*

### Construction of Genetic Risk Score

The genetic risk score for leukocyte telomere length is calculated in a weighted way (Ripatti et al., 2010; Guo et al., 2016; Zeng et al., 2019b).

(1) $$\text{GRS}=\sum_{j=1}^{17}G_{j}\,{\hat{\beta }}_{j}$$

where ${\hat{\beta }}_{j}$ is the estimated marginal SNP effect on the shorter leukocyte telomere length for the *j*th selected index SNP (e.g., Table 1) (Li et al., 2020). *G*_*j*_ is the individual-level genotype of the same SNP in the UK Biobank (Bycroft et al., 2018) or TCGA dataset (Hoadley et al., 2018) and is coded to be 0, 1, and 2, representing the number of effect allele. Following prior work (Zeng et al., 2019b), we do not directly rescale the GRS as its *p*-value would not be altered regardless of whether the GRS is scaled or not. We instead standardize the GRS so that its mean is zero and the variance is equal to 1.

### Two-Stage Regression Model in the UK Biobank and TCGA Using GRS

To link GRS with the risk of cancers from the UK Biobank (Table 2; Bycroft et al., 2018), we apply an additive logistic regression while adjusting for a set of available covariates (i.e., age, gender, smoke, drink, and BMI).

**TABLE 2:** Association between the genetic risk score (GRS) of leukocyte telomere length and the risk of 37 UK Biobank cancers.

Types of cancer	OR (95%CI)	*p*	FDR	Case	M/F	Age (years)
**Leukemia**	1.20 (1.02–1.41)	**0.025**	0.058	147	79/68	67.99 ± 8.17
Rectal cancer	1.10 (0.96–1.25)	0.165	0.193	231	134/97	70.64 ± 6.17
Tongue cancer	1.06 (0.88–1.29)	0.526	0.407	102	65/37	68.89 ± 7.35
Squamous cell carcinoma	1.04 (0.93–1.15)	0.514	0.401	332	168/164	70.89 ± 6.21
Testicular cancer	1.02 (0.95–1.11)	0.549	0.417	595	595/0	64.78 ± 8.02
Primary bone cancer	1.02 (0.81–1.29)	0.845	0.524	72	44/28	67.56 ± 8.02
Non-melanoma skin cancer	1.02 (0.93–1.12)	0.648	0.458	472	280/192	70.01 ± 6.97
Large bowel cancer/Colorectal cancer	1.02 (0.93–1.12)	0.739	0.490	440	260/180	71.54 ± 5.80
Rodent ulcer	1.01 (0.92–1.11)	0.893	0.538	437	203/234	70.50 ± 5.77
Esophageal cancer	1.01 (0.85–1.19)	0.946	0.552	137	110/27	71.98 ± 6.30
Cervical cancer	1.01 (0.95–1.06)	0.857	0.527	1273	0/1,273	66.16 ± 7.65
Non-Hodgkin’s lymphoma	0.99 (0.92–1.08)	0.945	0.552	593	355/238	69.40 ± 7.34
Pre-cancer cells cervix	0.99 (0.94–1.06)	0.922	0.546	1117	1/1,116	63.99 ± 7.98
Breast cancer	0.98 (0.96–1.01)	0.164	0.193	7330	37/7,293	70.08 ± 6.48
Colon cancer/sigmoid cancer	0.97 (0.92–1.04)	0.399	0.342	1055	631/424	72.30 ± 5.68
Uterine/endometrial cancer	0.95 (0.89–1.02)	0.176	0.200	752	0/752	71.27 ± 5.89
Ovarian cancer	0.95 (0.87–1.03)	0.222	0.224	512	0/512	69.11 ± 7.33
Brain cancer/primary malignant brain tumor	0.95 (0.80–1.13)	0.539	0.412	128	62/66	64.77 ± 8.81
**Prostate cancer**	0.94 (0.91–0.98)	**0.005**	**0.020**	2410	2,410/0	73.96 ± 4.08
Skin cancer	0.94 (0.88–1.00)	0.065	0.106	943	478/465	71.21 ± 6.38
**Basal cell carcinoma**	0.94 (0.90–0.97)	**0.001**	**0.010**	2916	1,206/1,710	70.02 ± 6.84
Stomach cancer	0.94 (0.77–1.14)	0.516	0.402	96	56/40	71.33 ± 6.22
**Malignant melanoma**	0.91 (0.88–0.95)	**4.57E-06**	**9.56E-05**	2526	1,031/1,495	68.95 ± 7.41
Larynx/throat cancer	0.91 (0.80–1.04)	0.161	0.191	228	190/38	71.12 ± 6.43
**Bladder cancer**	0.91 (0.84–0.98)	**0.010**	**0.030**	725	548/177	72.30 ± 5.87
Eye and/or adnexal cancer	0.90 (0.74–1.11)	0.325	0.297	95	44/51	68.82 ± 7.48
Thyroid cancer	0.90 (0.80–1.01)	0.067	0.108	293	52/241	67.27 ± 7.63
Small intestine/small bowel cancer	0.90 (0.76–1.06)	0.206	0.216	133	77/56	72.28 ± 5.55
**Hodgkin’s lymphoma/Hodgkin’s disease**	0.89 (0.79–0.99)	**0.033**	0.069	321	184/137	64.96 ± 8.13
Chronic myeloid leukemia	0.88 (0.71–1.10)	0.273	0.262	81	44/37	68.35 ± 7.98
Lung cancer	0.88 (0.74–1.06)	0.172	0.197	123	82/41	72.60 ± 5.70
**Kidney/renal cell cancer**	0.86 (0.78–0.95)	**0.003**	**0.017**	401	261/140	70.22 ± 6.36
Cancer of lip/mouth/pharynx/oral/cavity	0.86 (0.68–1.09)	0.213	0.220	69	43/26	70.42 ± 5.98
**Sarcoma/fibrosarcoma**	0.84 (0.72–0.98)	**0.028**	0.063	164	76/88	66.73 ± 7.58
**Chronic lymphocytic leukemia**	0.82 (0.71–0.94)	**0.005**	**0.020**	206	131/75	71.28 ± 6.28
Lymphoma	0.80 (0.64–1.01)	0.057	0.098	78	51/27	68.69 ± 8.27
**Multiple myeloma**	0.77 (0.63–0.93)	**0.006**	**0.021**	108	62/46	70.37 ± 7.08

*The cancers were sorted by the estimated odds ratios (ORs). *CI*, confidence internal; *p*, the original *p*-value; *FDR*, false discovery rate; *M*, male; *F*, female. In bold are significant (i.e., FDR < 0.05) or suggestive associations (i.e., p < 0.05).*

(2) $$\mathrm{logit}\,\left(\mu_{i}\right)={\text{GRS}}_{i}\times \theta +X_{i}^{T}\,\alpha$$

where *μ_*i*_* is the expectation of *y*_*i*_, with *y*_*i*_ = 1 or 0 representing the status of individual *i* with or without cancer; θ is the effect size of GRS; and ***X****_*i*_* is the vector of standardized covariates with effect sizes α. Of note, we assume that all of the entries in the first column of ***X*** are 1, representing the intercept term.

We next evaluate the effect of GRS on the mortality of cancers from TCGA (Table 3; Hoadley et al., 2018) with the Cox proportional hazards model (Cox, 1972) while controlling for available clinical covariates (i.e., age at diagnosis, gender, and stage).

**TABLE 3:** Association between the genetic risk score (GRS) of leukocyte telomere length and the mortality of 33 TCGA cancers.

Cancer	HR (95%CI)	*p*	FDR	*N*	Median survival time			M/F	Age at diagnosis (years)	Stage or grade (1/2/3/4/5)
					All	Event	Censor			
DLBC	2.24 (0.88–5.67)	0.090	0.317	42	31.85	19.83	32.4	19/23	55.33 ± 14.39	8/17/5/12
PCPG	2.16 (0.95–4.92)	0.068	0.283	178	25.28	15.08	25.6	78/100	47.30 ± 15.12	NA
**READ**	1.72 (1.09–2.73)	**0.020**	0.138	157	21.2	24.33	21.02	85/72	64.34 ± 11.67	30/51/51/25
UVM	1.47 (0.94–2.30)	0.092	0.320	79	25.77	19.68	27.37	44/35	61.68 ± 13.94	0/39/36/4
PRAD	1.44 (0.72–2.87)	0.306	0.610	501	30.8	29.17	30.87	501/0	60.93 ± 6.81	NA
**SARC**	1.29 (1.06–1.58)	**0.011**	0.138	260	31.77	21.6	36.4	119/141	60.80 ± 14.61	NA
ESCA	1.28 (0.99–1.66)	0.063	0.274	162	13.57	13.38	13.57	137/25	62.40 ± 11.74	18/79/56/9
TGCT	1.23 (0.10–15.59)	0.870	0.816	81	37.53	116.48	37.53	81/0	32.85 ± 10.18	55/12/14/0
**SKCM**	1.19 (1.03–1.37)	**0.018**	0.138	411	33.2	31.93	34.5	256/155	58.82 ± 15.51	77/140/171/23
KICH	1.17 (0.46–2.99)	0.743	0.792	65	74.93	28.5	90.43	38/27	51.15 ± 13.99	20/25/14/6
CESC	1.14 (0.90–1.45)	0.274	0.583	295	21.27	20.23	23.12	0/295	47.88 ± 13.47	160/69/46/20
THCA	1.12 (0.66–1.89)	0.676	0.776	503	31.67	34.03	31.47	136/367	47.28 ± 15.78	284/52/113/54
BRCA	1.11 (0.93–1.32)	0.258	0.569	924	26.38	44.13	24.23	0/924	58.84 ± 13.14	156/523/219/14/12
LUSC	1.06 (0.93–1.21)	0.378	0.659	487	21.77	18.13	24.58	359/128	67.31 ± 8.58	239/157/84/7
MESO	1.05 (0.82–1.35)	0.705	0.783	86	17.1	15.23	38.93	70/16	63.08 ± 9.72	10/16/44/16
LUAD	1.04 (0.90–1.21)	0.584	0.749	503	21.87	20.47	22.33	232/271	65.16 ± 10.07	277/121/80/25
UCEC	1.04 (0.84–1.28)	0.742	0.791	546	30.47	23.63	32.2	0/546	63.99 ± 11.13	338/52/127/29
KIRC	1.04 (0.90–1.20)	0.639	0.766	532	39.2	27.35	48.1	342/190	60.57 ± 12.07	267/57/125/83
HNSC	1.03 (0.90–1.19)	0.655	0.770	450	21.37	14.08	27.4	324/126	60.90 ± 12.13	27/73/82/268
LIHC	1.00 (0.83–1.21)	0.963	0.831	350	19.43	13.67	21.47	239/111	59.03 ± 13.30	174/86/85/5
GBM	1.00 (0.91–1.10)	0.949	0.829	595	12.27	12.7	8.67	364/231	57.87 ± 14.41	NA
ACC	0.99 (0.68–1.44)	0.967	0.832	88	37.93	18.38	48.45	29/59	47.07 ± 16.43	9/43/18/18
OV	0.98 (0.88–1.08)	0.629	0.763	569	33.57	35.77	28.57	0/569	59.71 ± 11.46	16/30/437/86
PAAD	0.97 (0.80–1.19)	0.792	0.802	182	15.55	13.13	16.92	100/82	64.92 ± 11.06	21/152/4/5
STAD	0.96 (0.83–1.11)	0.576	0.746	407	14.53	11.6	18.87	260/147	65.37 ± 10.70	55/128/181/43
BLCA	0.96 (0.82–1.12)	0.576	0.746	411	17.87	13.68	21.27	303/108	68.10 ± 10.58	3/131/141/136
COAD	0.93 (0.76–1.13)	0.448	0.696	458	22.32	13.47	24.33	239/219	67.03 ± 13.06	79/183/131/65
LGG	0.89 (0.73–1.09)	0.270	0.580	512	22.47	27.13	20.97	284/228	42.99 ± 13.34	0/247/265/0
LAML	0.89 (0.73–1.09)	0.252	0.563	186	12.17	9.1	23.3	102/84	55.53 ± 16.06	NA
UCS	0.85 (0.59–1.23)	0.395	0.669	56	20.25	16.72	27.6	0/56	69.38 ± 8.89	21/5/20/10
THYM	0.85 (0.41–1.76)	0.654	0.770	121	41.77	28.43	42.33	62/59	58.37 ± 12.94	37/61/15/8
**KIRP**	0.66 (0.47–0.93)	**0.019**	0.138	257	24.67	20.8	25.37	190/67	61.50 ± 12.03	171/20/51/15
CHOL	0.64 (0.38–1.08)	0.097	0.331	36	21.5	16.67	31.42	16/20	63.03 ± 12.67	19/9/1/7

*The cancers were sorted by the estimated hazard ratios (HRs). *CI*, confidence internal; *p*, the original *p*-value; *FDR*, false discovery rate; *M*, male; *F*, female. Cancer types: *DLBC*, lymphoid neoplasm diffuse large B-cell lymphoma; *PCPG*, pheochromocytoma and paraganglioma; *READ*, rectum adenocarcinoma; *UVM*, uveal melanoma; *PRAD*, prostate adenocarcinoma; *SARC*, sarcoma; *ESCA*, esophageal carcinoma; *TGCT*, testicular germ cell tumor; *SKCM*, skin cutaneous melanoma; *KICH*, kidney chromophobe; *CESC*, cervical squamous cell carcinoma and endocervical adenocarcinoma; *THCA*, thyroid carcinoma; *BRCA*, breast invasive carcinoma; *LUSC*, lung squamous cell carcinoma; *MESO*, mesothelioma; *LUAD*, lung adenocarcinoma; *UCEC*, uterine corpus endometrial carcinoma; *KIRC*, kidney renal clear cell carcinoma; *HNSC*, head and neck squamous cell carcinoma; *LIHC*, liver hepatocellular carcinoma; *GBM*, glioblastoma multiforme; *ACC*, adrenocortical carcinoma; *OV*, ovarian serous cystadenocarcinoma; *PAAD*, pancreatic adenocarcinoma; *STAD*, stomach adenocarcinoma; *BLCA*, bladder urothelial carcinoma; *COAD*, colon adenocarcinoma; LGG, brain lower grade glioma; *LAML*, acute myeloid leukemia; *UCS*, uterine carcinosarcoma; *THYM*, thymoma; *KIRP*, kidney renal papillary cell carcinoma; *CHOL*, cholangiocarcinoma. In bold are suggestive associations (i.e., p < 0.05).*

(3) $$h\,\left(t_{i}|{\text{GRS}}_{i},X_{i}\right)=h_{0}\,\left(t_{i}\right)\,e^{{\text{GRS}}_{i}\times \theta +X_{i}^{T}\,\alpha }$$

where *t*_*i*_ is the observed survival time and *h*_0_(*t*) is an arbitrary baseline hazard function. Cancer-specific covariates are considered for some cancers in TCGA [e.g., the status of estrogen and progesterone receptors for breast invasive carcinoma (BRCA)]. In the logistic or Cox model, we are mainly interested in estimating θ and testing for the null hypothesis *H*_0_: θ = 0. We further examine the interaction effect between GRS and each of the clinical covariates (e.g., GRS × gender) if GRS is detected to be associated with some cancer.

### Two-Sample MR Analysis

Besides the GRS method, we also perform the two-sample MR analysis to estimate the causal effect of leukocyte telomere length on cancers in the UK Biobank using summary statistics (Sudlow et al., 2015). In observational studies, MR is a flexible approach for causal inference to avert confounding and reverse causality (Zeng et al., 2019a; Yu et al., 2020). In brief, we estimate the causal effect of leukocyte telomere length (again, denoted as θ) relying on all the available instrumental variables (Table 1) through the commonly employed inverse-variance weighted (IVW) method (Burgess et al., 2017; Hartwig et al., 2017).

(4) $$\begin{array}{c} \hat{\theta }=\frac{1}{\sum_{j=1}^{17}\text{var}\,{\left({\hat{\beta }}_{j}^{\text{Y}}\right)}^{-1}\,{\left({\hat{\beta }}_{j}^{\text{X}}\right)}^{2}}\,\sum_{j=1}^{17}\text{var}\,{\left({\hat{\beta }}_{j}^{\text{Y}}\right)}^{-1}\,{\hat{\beta }}_{j}^{\text{Y}}\,{\hat{\beta }}_{j}^{\text{X}}\\ \text{var}\,\left(\hat{\theta }\right)=\frac{1}{\sum_{j=1}^{17}\text{var}\,{\left({\hat{\beta }}_{j}^{\text{Y}}\right)}^{-1}\,{\left({\hat{\beta }}_{j}^{\text{X}}\right)}^{2}} \end{array}$$

where ${\hat{\beta }}_{j}^{\text{X}}$ and $\text{var}\,\left({\hat{\beta }}_{j}^{\text{X}}\right)$ are the effect size and the variance, respectively, of the instrumental variable *j* for the exposure X (i.e., leukocyte telomere length; Li et al., 2020), and ${\hat{\beta }}_{j}^{\text{Y}}$ and $\text{var}\,\left({\hat{\beta }}_{j}^{\text{Y}}\right)$ are the effect size and the variance, respectively, for the same instrumental variable *j* on the outcome Y (i.e., cancer in the UK Biobank; Sudlow et al., 2015).

To guarantee the validity of our MR analysis, before the formal analysis, we examine the pleiotropic effects of instruments by removing index SNPs that may be potentially related to individual cancers if the Bonferroni-adjusted *p*-values are less than 0.05. We also conduct a series of sensitivity analyses: (i) weighted median-based (Bowden et al., 2016b) and maximum likelihood methods (Burgess et al., 2013), which are robust when some instrumental variables might be invalid; (ii) MR-Egger regression (Bowden et al., 2016a; Burgess and Thompson, 2017), which guards against horizontal pleiotropic effects; and (iii) leave-one-out (LOO) analysis (Noyce et al., 2017) and Mendelian randomization pleiotropy residual sum and outlier (MR-PRESSO) test (Verbanck et al., 2018) to examine potential instrumental outliers.

### UK Biobank and TCGA Cancer Datasets

The UK Biobank dataset consists of approximately 500,000 individuals (Bycroft et al., 2018). We selected age, gender, smoke, drink, and BMI as covariates and originally chose 79 self-reported cancers up to 337,198 independent individuals (28,820 cases and 308,378 controls) of European ancestry, but only included cancers with at least 60 cases (to some extent, this cutoff value was used arbitrarily) and treated cancer-free individuals to be controls. Finally, a total of 37 cancers were left up to 335,036 individuals (27,641 cases for various cancers and 307,395 shared cancer-free controls after removing individuals with missing values). The genotypes were provided by the UK Biobank after the research application was approved. However, we can only obtain 15 SNPs because two were missing (i.e., rs3219104 on *PARP1* and rs55749605 on *SENP7*) in the UK Biobank. In addition, because summary-level statistics are necessary for the two-sample MR analysis, herein we can only consider 28 cancers from the UK Biobank (*n* = 420,473) (Sudlow et al., 2015; Supplementary Table S6). The summary statistics of these cancers were obtained from https://pan.ukbb.broadinstitute.org/.

Then, we obtained the survival and clinical information of 33 cancers from TCGA (Hoadley et al., 2018). We selected the overall survival time and status as the outcome and primarily included age at diagnosis, gender, and pathologic tumor stage as covariates because many other important clinical covariates were missing for most of the patients. When the pathologic tumor stage cannot be available, we instead employed the clinical stage (i.e., for CESC, DLBC, OV, THYM, UCEC, and UCS) or histological grade (i.e., for LGG). It needs to be stated that all three stage variables were missing in five cancers (i.e., GBM, LAML, PCPG, PRAD, and SARC). For each cancer, we only kept samples from the primary cancer tissue and excluded those with missing values in clinical covariates. More details about these TCGA cancers are demonstrated in Table 3 and Supplementary Table S3. For each cancer, we filtered out SNPs that had a missingness rate >0.95 across individuals, genotype calling rate <0.95, minor allele frequency (MAF) > 0.01, or Hardy–Weinberg equilibrium (HWE) *p*-value < 10^–4^. We next performed an imputation procedure by first phasing the genotypes with SHAPEIT (Delaneau et al., 2013), then imputed the SNPs based on the Haplotype Reference Consortium panel (McCarthy et al., 2016) on the Michigan Imputation Server using minimac3 (Das et al., 2016). The filtering procedure for the imputed genotypes included an HWE *p*-value < 10^–4^, a genotype call rate <95%, a MAF < 0.01, and an imputation score <0.30. After the imputation of genotypes, all of the 17 SNPs were yielded in TCGA.

### Power Evaluation

Finally, we performed power calculation to detect a non-zero causal effect for GRS with regards to cancers based on the UK Biobank and TCGA datasets. Firstly, we simulated genotypes for 17 independent SNPs with varying MAFs (Table 1) and then calculated the GRS. Two independent covariates (i.e., one was binary and the other was continuous) were also included, with each having an effect size of 0.5. We generated a case–control variable *y* with the probability of exp(η)/(1 + exp(η)) and η = GRS × θ + 0.5*X*_1_ + 0.5*X*_2_. We created 2,000,000 individuals to be the population and then randomly sampled 50 (or 100 and 150) cases and 300,000 controls (as well as their GRS and covariates) to be a subset for the final simulation analysis.

Secondly, to simulate survival datasets, we first generated genotypes and calculated the GRS in the same way as described above. Again, two independent covariates were included, with each having an effect size of 0.5. Then, we employed the inverse probability method (Bender et al., 2005) to create survival time which followed a Weibull distribution, with the shape parameter being 1 and the scale parameter being 0.01. The location parameter of this Weibull distribution was determined by the GRS and the two covariates [i.e., *μ* = exp(η), with η = GRS × θ + 0.5*X*_1_ + 0.5*X*_2_]. The censored rate was fixed to be 50% in a random manner (the high censored rate corresponded to a similar situation observed in the TCGA cancer dataset). The sample size varied from 100, 300, to 500.

In both simulations, the effect size of GRS θ was set to 0.05, 0.10, or 0.20, approximately corresponding to odds ratios (ORs) [or hazard ratio (HR)] of 1.05, 1.10, and 1.20. The simulation was repeated 1,000 times, and the power calculated by the proportion of the *p*-value of GRS was less than 1.67E-3, approximately equal to the significance level after the Bonferroni correction of 30 types of cancers.

Throughout our study, we utilized the R software (version 3.6.1) to implement all the analyses. The association was declared to be statistically significant if the false discovery rate (FDR) is <0.05 (Benjamini and Hochberg, 1995), while the association was deemed to be suggestive if the unadjusted *p*-value is <0.05.

## Results

### Association Between GRS and UK Biobank Cancers

The 17 selected index SNPs collectively explain about 1.37% phenotypic variance of leukocyte telomere length, and all the F statistics are above 10 (ranging from 27.9 to 205.4, with an average of 63.3) (Table 1), largely ruling out the possibility of weak instrument bias (Cragg and Donald, 1993; Burgess et al., 2017; Zeng and Zhou, 2019a). Based on the constructed GRS, we first investigate the association between leukocyte telomere length and the risk of UK Biobank cancers (Table 2). We detect that the GRS of leukocyte telomere length is significantly associated with a decreased risk of seven types of cancers (Table 2), including multiple myeloma [OR = 0.77, 95% confidence interval (CI) = 0.63–0.93, FDR = 0.021], chronic lymphocytic leukemia (OR = 0.82, 95%CI = 0.71–0.94, FDR = 0.020), kidney/renal cell cancer (OR = 0.86, 95%CI = 0.78–0.95, FDR = 0.017), bladder cancer (OR = 0.91, 95%CI = 0.84–0.98, FDR = 0.030), malignant melanoma (OR = 0.91, 95%CI = 0.88–0.95, FDR = 9.56E-05), basal cell carcinoma (OR = 0.94, 95%CI = 0.90–0.97, FDR = 0.010), and prostate cancer (OR = 0.94, 95%CI = 0.91–0.98, FDR = 0.020). Suggestive associations are observed for two types of cancers including sarcoma/fibrosarcoma (OR = 0.84, 95%CI = 0.72–0.98, FDR = 0.063) and Hodgkin’s lymphoma/Hodgkin’s disease (OR = 0.89, 95%CI = 0.79–0.99, FDR = 0.069). In addition, we discover that the GRS of leukocyte telomere length is also marginally related to an increased risk of leukemia (OR = 1.20, 95%CI = 1.02–1.41, FDR = 0.058).

We further examine the interaction effect of GRS and one of the covariates (e.g., age, gender, smoke, drink, or BMI) for each of the 10 cancers. We observe that the interaction term is statistically significant between smoke and GRS for sarcoma/fibrosarcoma (OR = 0.83, 95%CI = 0.71–0.97) as well as between drink and GRS for leukemia (OR = 0.82, 95%CI = 0.69–0.97) (Supplementary Table S4).

### Association Between GRS and TCGA Cancers

We now examine the effect size of GRS on 33 TCGA cancers through the Cox proportional hazards model. We observe suggestive evidence that the GRS of leukocyte telomere length is related to a higher death hazard of READ (HR = 1.72, 95%CI = 1.09–2.73, *p* = 0.020), SARC (HR = 1.29, 95%CI = 1.06–1.58, *p* = 0.011), and SKCM (HR = 1.19, 95%CI = 1.03–1.37, *p* = 0.018) and is associated with a lower death hazard of KIRP (HR = 0.66, 95%CI = 0.47–0.93, *p* = 0.019), suggesting that a genetically decreased leukocyte telomere length can lead to a worse overall survival of READ, SARC, and SKCM while can result in a better overall survival of KIRP. However, all these associations become non-significant after accounting for multiple comparisons (FDR > 0.05). Neither suggestive nor significant associations are identified between GRS and the remaining cancers (Table 3). We further examine the interaction effect of GRS and each of the covariates (e.g., age at diagnosis, gender, or stage) for each of the four cancers. We do not identify any statistically significant interactions (Supplementary Table S5).

### Association Between Leukocyte Telomere Length and UK Biobank Cancers Using the Two-Sample MR

With the selected 17 instrumental variables, we further perform MR analysis to investigate the causal effect of leukocyte telomere length on each of the 28 cancers from the UK Biobank. As no evidence of effect heterogeneity is presented across instruments (all the *p*-values for the Cochran’s *Q* test are greater than 0.05), thus, only the results estimated *via* the fixed-effects IVW method are displayed below. Among the 28 cancers, we identify that leukocyte telomere length is associated with a decreased risk of nine cancers (Supplementary Table S6), including basal cell carcinoma, malignant melanoma, skin cancer, bladder cancer, kidney/renal cell cancer, Hodgkin’s lymphoma/Hodgkin’s disease, thyroid cancer, chronic lymphocytic leukemia, and multiple myeloma. We also observe that leukocyte telomere length is associated with an increased risk of leukemia (Supplementary Table S6).

**FIGURE 1:**
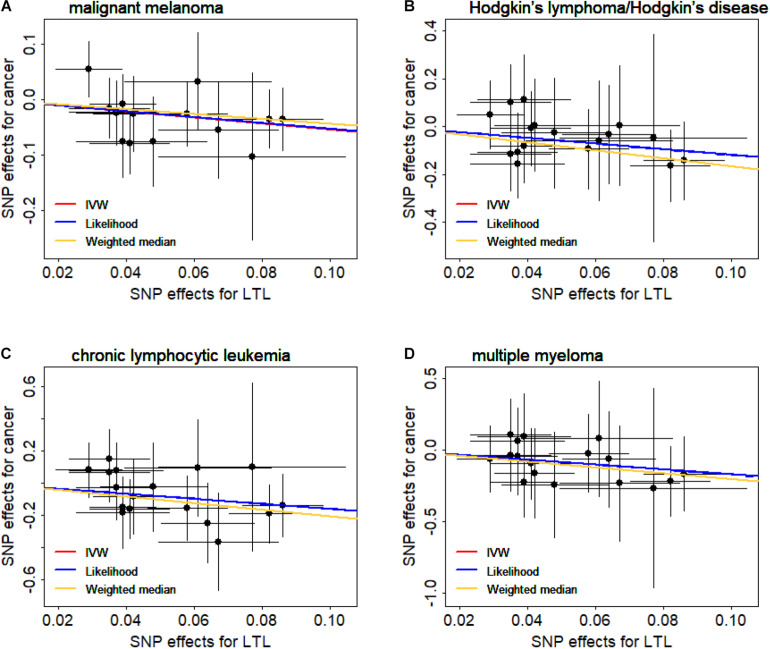
Relationship between the single nucleotide polymorphism (SNP) effect sizes of leukocyte telomere length (LTL) (*x*-axis) and the corresponding effect sizes of cancer (*y*-axis). **(A)** Malignant melanoma. **(B)** Hodgkin’s lymphoma/Hodgkin’s disease. **(C)** Chronic lymphocytic leukemia. **(D)** Multiple myeloma. In the plot, horizontal/vertical lines represent the 95% confidence interval.

We now validate the observed causal associations shown above through various sensitivity analyses (Supplementary Table S6). Here, we focus on the associations that are significant in all sensitivity analyses (i.e., *P*_*Weighted  median*_ and *P*_*Likelihood*_ < 0.05) and have no horizontal pleiotropic effects (i.e., *P*_*Egger–intercept*_ > 0.05). Then, four types of cancers are left, including malignant melanoma (OR = 0.58, 95%CI = 0.44–0.79, FDR = 0.004), Hodgkin’s lymphoma/Hodgkin’s disease (OR = 0.30, 95%CI = 0.13–0.69, FDR = 0.008), chronic lymphocytic leukemia (OR = 0.20, 95%CI = 0.08–0.54, FDR = 0.004), and multiple myeloma (OR = 0.18, 95%CI = 0.05–0.66, FDR = 0.018). Of note is that both the weighted median method and the maximum likelihood method generate consistent causal effect estimates compared with the IVW method (Supplementary Table S6). In addition, we create scatter plots for the SNP effect sizes of leukocyte telomere length and these four cancers (Figure 1); we find that no instruments may be potential outliers. The finding is also supported by MR-PRESSO, which displays the absence of instrument outliers at the significance level of 0.05.

To further examine whether a single instrumental variable may strongly influence the causal effects of leukocyte telomere length on these four cancers, we performed the LOO analysis. Again, the LOO analysis results demonstrate that none of the 17 instruments can substantially impact the estimated casual effect. Therefore, we can conclude that it is likely that a shorter leukocyte telomere length can decrease the risk of malignant melanoma, Hodgkin’s lymphoma/Hodgkin’s disease, chronic lymphocytic leukemia, and multiple myeloma. This finding here is also consistent with the results derived by the GRS regression above.

### Power Calculation for the Association Between GRS and Cancers in the UK Biobank/TCGA Datasets

**FIGURE 2:**
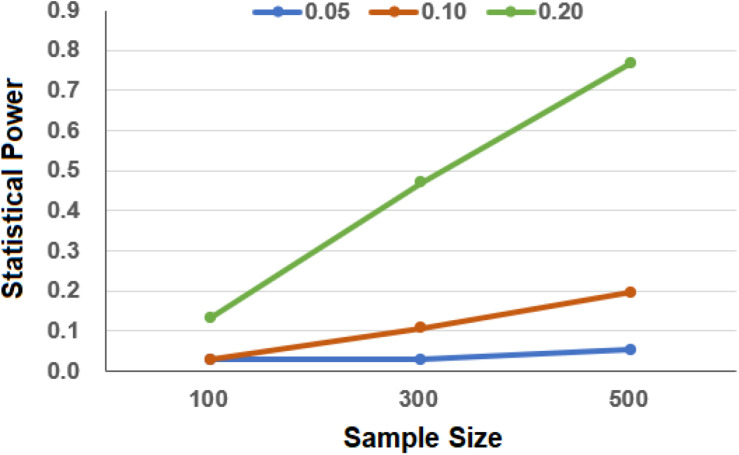
Estimated power in the simulation to evaluate the association between genetic risk score (GRS) and cancers in The Cancer Genome Atlas (TCGA). In the simulation, the effect sizes of GRS were set to 0.05, 0.10, and 0.20 and the sample sizes of cancer were set to 100, 300, and 500.

In terms of our simulations, we have sufficient power to detect the association in the UK Biobank as the total sample size is large, although only a few of the cancer cases are included. Specifically, we observe that the estimated power approaches 100% even when the number of cases is only 50 and the OR is only 1.05. In contrast, due to the relatively weak effect size and small sample size in the simulated TCGA cancer dataset, under our simulation settings, we have only low to moderate power to detect the association between GRS and the survival risk of cancer (Figure 2). For example, when the sample size is 300, the statistical power is only 3.0 or 10.7% when the HR was set to be 1.05 or 1.10. As can be expected, the power improves with the increase in the sample sizes and effect sizes.

## Discussion

### Summary of the Results of the Present Study

The main objective of our study was to investigate whether there existed associations between genetically predicted leukocyte telomere length and various types of cancers. To achieve this, we first constructed the GRS of leukocyte telomere length based on associated SNPs from a large-scale GWAS and evaluated the effect of GRS on the risk and mortality of cancers. We found statistical evidence supporting the existence of associations between GRS and cancers in the UK Biobank and TCGA. Briefly, based on the GRS, a shorter leukocyte telomere length was identified to be associated with the decreased risk of some cancers (i.e., multiple myeloma, chronic lymphocytic leukemia, kidney/renal cell cancer, bladder cancer, malignant melanoma, basal cell carcinoma, prostate cancer, sarcoma/fibrosarcoma, and Hodgkin’s lymphoma/Hodgkin’s disease) as well as related to the decreased mortality of KIRP. In addition, inverse associations were observed for shorter leukocyte telomere length on the risk of leukemia as well as on the mortality of READ, SARC, and SKCM. The results of the MR analysis also supported the existence of an association between leukocyte telomere length and various cancers, including malignant melanoma, Hodgkin’s lymphoma/Hodgkin’s disease, chronic lymphocytic leukemia, and multiple myeloma. The diverse associations between leukocyte telomere length and cancers may in part reflect the different carcinogenic mechanisms acted by telomere in specific cancer types, further suggesting that telomere length is a valuable indicator of cancer risk and prognosis.

### Discoveries Combined With the Previous Study

We found that the observed associations between leukocyte telomere length and cancers in the present study (i.e., multiple myeloma, chronic lymphocytic leukemia, kidney/renal cell cancer, bladder cancer, malignant melanoma, basal cell carcinoma, and prostate cancer) are greatly consistent with prior findings obtained in terms of MR (Supplementary Table S1; Zhang et al., 2015; Ojha et al., 2016; Haycock et al., 2017; Machiela et al., 2017; Li et al., 2020; Went et al., 2020). Particularly, several previous studies demonstrated that a shorter telomere length was associated with a decreased lung cancer risk or mortality and that the association was present in adenocarcinoma while absent in squamous cell carcinoma (Supplementary Table S1; Zhang et al., 2015; Haycock et al., 2017; Kachuri et al., 2018; Yuan et al., 2018), which may be attributed to the discrepancy in the biological characteristics of various subtypes of lung cancer. In the present study, inconsistent correlations were also identified within different subtypes of cancer. For example, we discovered that leukocyte telomere length had an opposite effect on the risk of leukemia and chronic lymphocytic leukemia. However, we observed that leukocyte telomere length displayed similar effects on the risk of malignant melanoma and basal cell carcinoma. These findings suggest that leukocyte telomere may influence the risk or mortality of cancer in a histologic way and also emphasize the unique roles of leukocyte telomere in the development of cancers.

Although the molecular mechanism remains unclear, some prior studies implied that both short and long telomere length played an important role in the etiology of cancers (Cui et al., 2012; Cheng et al., 2017; Nelson and Codd, 2020). Cells with longer telomere lengths have greater proliferative potential and more probability of accruing mutations (Hanahan and Weinberg, 2011); therefore, telomere shortening is generally considered to be a protective mechanism against tumorigenesis (Rode et al., 2016; Zhang et al., 2017; Kuo et al., 2019). However, it has been proposed that telomere shortening can generally give rise to end-to-end chromosome fusions and attenuates DNA damage response, thus increasing genomic instability and finally initiating carcinogenesis (Wu et al., 2003). These findings indicate that telomere plays a dual role in cancer development, and such role seems to depend on the types of cancers and the balance of the proliferation and senescence of cells in cancers.

### Strengths and Limitations of Our Study

One advantage of our study is that more than 50 diverse types of cancers were investigated; it is thus feasible to undertake a systematic evaluation in the present analysis. In addition, methodologically, the GRS analysis can be viewed to be a two-stage regression model within the framework of instrumental variable-based causal inference (Baum et al., 2003; Hernán and Robins, 2006; Zeng et al., 2019a). Specifically, leukocyte telomere length is the exposure of interest and the associated SNPs are the carefully selected instrumental variables which are supposed to satisfy the necessary assumptions of instruments (Lawlor et al., 2008; Sheehan et al., 2008; Zeng et al., 2019a; Zeng and Zhou, 2019a, b). In the first stage, the effect size of each instrumental variable is estimated with an external large-scale GWAS dataset; in the second stage, the influence of leukocyte telomere length on various cancers is assessed based on the genetically determined leukocyte telomere length which is predicted with the chosen instrumental variables. Therefore, in terms of the principle of instrumental variable inference, the estimated effect of GRS can be interpreted as causal. In this sense, besides the MR method, we are actually investigating the causal association between leukocyte telomere length and cancers by constructing a GRS.

Finally, some shortcomings of this study should also be mentioned. Firstly, the majority of the individuals of the UK Biobank and TCGA were of European ancestry, so our results may not be applicable to other populations. Secondly, in our study, telomere length measured in blood leukocytes was employed and not in all cell types *in vivo*; however, leukocyte telomere length was demonstrated to be highly correlated with that in cells from other tissues (Friedrich et al., 2000; Wilson et al., 2008; Butt et al., 2010). Thirdly, as described before, the effect sizes of leukocyte telomere length on the mortality of TCGA cancers were only suggestive and the sample size of these cancers was not sufficiently large to maintain high power to detect weak associations. Therefore, further investigations with a larger sample size are required to validate our results.

## Conclusion

Our study reveals that telomere played diverse roles in different types of cancers; however, further validations in large-scale prospective studies and deeper investigations of the biologic mechanisms are warranted.

## Data Availability Statement

The datasets presented in this study can be found in online repositories. The names of the repository/repositories and accession number(s) can be found in the article/Supplementary Material.

## Author Contributions

PZ conceived the idea for the study. PZ, YW, XZ, SH, and HZ obtained the data. PZ and YG cleared up the datasets, performed the data analyses, and drafted the manuscript. PZ, YG, and YW interpreted the results of the data analyses. All authors approved the manuscript and provided relevant suggestions.

## Conflict of Interest

The authors declare that the research was conducted in the absence of any commercial or financial relationships that could be construed as a potential conflict of interest.

## References

[B1] Baum C. F. Schaffer M. E. Stillman S. (2003). Instrumental variables and GMM: estimation and testing. *Stat. J.* 3 1–31. 10.1177/1536867x0300300101

[B2] Belsky D. W. Moffitt T. E. Sugden K. Williams B. Houts R. McCarthy J. (2013). Development and evaluation of a genetic risk score for obesity. *Biodemogr. Soc. Biol.* 59 85–100. 10.1080/19485565.2013.774628 23701538 PMC3671353

[B3] Bender R. Augustin T. Blettner M. (2005). Generating survival times to simulate Cox proportional hazards models. *Stat. Med.* 24 1713–1723. 10.1002/sim.2059 15724232

[B4] Benjamini Y. Hochberg Y. (1995). Controlling the false discovery rate: a practical and powerful approach to multiple testing. *J. R. Statist. Soc. Ser. B* 57 289–300. 10.1111/j.2517-6161.1995.tb02031.x

[B5] Bogdan R. Baranger D. A. A. Agrawal A. (2018). Polygenic risk scores in clinical psychology: bridging genomic risk to individual differences. *Ann. Rev. Clin. Psychol.* 14 119–157. 10.1146/annurev-clinpsy-050817-084847 29579395 PMC7772939

[B6] Bowden J. Del Greco M. F. Minelli C. Smith G. D. Sheehan N. A. Thompson J. R. (2016a). Assessing the suitability of summary data for two-sample mendelian randomization analyses using MR-Egger regression: the role of the I-2 statistic. *Int. J. Epidemiol.* 45 1961–1974. 10.1093/ije/dyw220 27616674 PMC5446088

[B7] Bowden J. Smith G. D. Haycock P. C. Burgess S. (2016b). Consistent estimation in mendelian randomization with some invalid instruments using a weighted median estimator. *Genet. Epidemiol.* 40 304–314. 10.1002/gepi.21965 27061298 PMC4849733

[B8] Burgess S. Butterworth A. Thompson S. G. (2013). Mendelian randomization analysis with multiple genetic variants using summarized data. *Genet. Epidemiol.* 37 658–665. 10.1002/gepi.21758 24114802 PMC4377079

[B9] Burgess S. Small D. S. Thompson S. G. (2017). A review of instrumental variable estimators for Mendelian randomization. *Stat. Methods Med. Res.* 26 2333–2355. 10.1177/0962280215597579 26282889 PMC5642006

[B10] Burgess S. Thompson S. G. (2012). Improving bias and coverage in instrumental variable analysis with weak instruments for continuous and binary outcomes. *Stat. Med.* 31 1582–1600. 10.1002/sim.4498 22374818

[B11] Burgess S. Thompson S. G. (2017). Interpreting findings from Mendelian randomization using the MR-Egger method. *Eur. J. Epidemiol.* 32 377–389. 10.1007/s10654-017-0255-x 28527048 PMC5506233

[B12] Burgess S. Thompson S. G. Collaboration C. C. G. (2011). Avoiding bias from weak instruments in Mendelian randomization studies. *Int. J. Epidemiol.* 40 755–764. 10.1093/ije/dyr036 21414999

[B13] Butt H. Z. Atturu G. London N. J. Sayers R. D. Bown M. J. (2010). Telomere length dynamics in vascular disease: a review. *Eur. J. Vasc. Endovasc. Surg.* 40 17–26. 10.1016/j.ejvs.2010.04.012 20547081

[B14] Bycroft C. Freeman C. Petkova D. Band G. Elliott L. T. Sharp K. (2018). The UK Biobank resource with deep phenotyping and genomic data. *Nature* 562 203–209. 10.1038/s41586-018-0579-z 30305743 PMC6786975

[B15] Cheng Y. Yu C. Huang M. Du F. Song C. Ma Z. (2017). Genetic association of telomere length with hepatocellular carcinoma risk: a Mendelian randomization analysis. *Cancer Epidemiol.* 50 (Pt A), 39–45. 10.1016/j.canep.2017.07.011 28797893

[B16] Codd V. Nelson C. P. Albrecht E. Mangino M. Deelen J. Buxton J. L. (2013). Identification of seven loci affecting mean telomere length and their association with disease. *Nat. Genet.* 45 422–427. 10.1038/ng.2528 23535734 PMC4006270

[B17] Cox D. R. (1972). Regression models and life-tables. *J. R. Statist. Soc. Ser.* 34 187–220.

[B18] Cragg J. G. Donald S. G. (1993). Testing identifiability and specification in instrumental variable models. *Economet. Theor.* 9 222–240. 10.1017/s0266466600007519

[B19] Cui Y. Cai Q. Y. Qu S. M. Chow W. H. Wen W. Q. Xiang Y. B. (2012). Association of leukocyte telomere length with colorectal cancer risk: nested case-control findings from the shanghai women’s health study. *Cancer Epidemiol. Biomark. Prevent.* 21 1807–1813. 10.1158/1055-9965.Epi-12-0657 22911335 PMC3467322

[B20] Das S. Forer L. Schonherr S. Sidore C. Locke A. E. Kwong A. (2016). Next-generation genotype imputation service and methods. *Nat. Genet.* 48 1284–1287. 10.1038/ng.3656 27571263 PMC5157836

[B21] De La Vega F. M. Bustamante C. D. (2018). Polygenic risk scores: a biased prediction? *Genome Med.* 10:100. 10.1186/s13073-018-0610-x 30591078 PMC6309089

[B22] de Lange T. (2005). Shelterin: the protein complex that shapes and safeguards human telomeres. *Genes Dev.* 19 2100–2110. 10.1101/gad.1346005 16166375

[B23] Delaneau O. Zagury J. F. Marchini J. (2013). Improved whole-chromosome phasing for disease and population genetic studies. *Nat. Methods* 10 5–6. 10.1038/nmeth.2307 23269371

[B24] Dorajoo R. Chang X. Gurung R. L. Li Z. Wang L. Wang R. (2019). Loci for human leukocyte telomere length in the Singaporean Chinese population and trans-ethnic genetic studies. *Nat. Commun.* 10:2491 10.1038/s41467-019-10443-10442 PMC6554354 31171785

[B25] Dudbridge F. Visscher P. Brown M. McCarthy M. Yang J. Wray N. (2013). Power and predictive accuracy of polygenic risk scores. *PLoS Genet.* 9:e1003348. 10.1371/journal.pgen.1003348 23555274 PMC3605113

[B26] Duncan L. Shen H. Gelaye B. Meijsen J. Ressler K. Feldman M. (2019). Analysis of polygenic risk score usage and performance in diverse human populations. *Nat. Commun.* 10:3328 10.1038/s41467-019-11112-11110 PMC6658471 31346163

[B27] Eusden J. Lewis C. M. O’Reilly P. F. (2015). PRSice: polygenic risk score software. *Bioinformatics* 31 1466–1468. 10.1093/bioinformatics/btu848 25550326 PMC4410663

[B28] Friedrich U. Griese E. Schwab M. Fritz P. Thon K. Klotz U. (2000). Telomere length in different tissues of elderly patients. *Mech. Age. Dev.* 119 89–99. 10.1016/s0047-6374(00)00173-171 11080530

[B29] Goldman D. (2017). Polygenic risk scores in psychiatry. *Biol. Psychiatry* 82 698–699. 10.1016/j.biopsych.2017.09.002 29031918

[B30] Gu J. A. Chen M. Shete S. Amos C. I. Kamat A. Ye Y. Q. (2011). A genome-wide association study identifies a locus on chromosome 14q21 as a predictor of leukocyte telomere length and as a marker of susceptibility for bladder cancer. *Cancer Prevent. Res.* 4 514–521. 10.1158/1940-6207.Capr-11-0063 21460395 PMC3076128

[B31] Guo Y. Andersen S. W. Shu X. O. Michailidou K. Bolla M. K. Wang Q. (2016). Genetically predicted body mass index and breast cancer risk: mendelian randomization analyses of data from 145,000 women of European descent. *PLoS Med.* 13:2105. 10.1371/journal.pmed.1002105 27551723 PMC4995025

[B32] Hackett J. A. Greider C. W. (2002). Balancing instability: dual roles for telomerase and telomere dysfunction in tumorigenesis. *Oncogene* 21 619–626. 10.1038/sj.onc.1205061 11850787

[B33] Hanahan D. Weinberg R. A. (2011). Hallmarks of cancer: the next generation. *Cell* 144 646–674. 10.1016/j.cell.2011.02.013 21376230

[B34] Hartwig F. P. Davey Smith G. Bowden J. (2017). Robust inference in summary data Mendelian randomization via the zero modal pleiotropy assumption. *Int. J. Epidemiol.* 46 1985–1998. 10.1093/ije/dyx102 29040600 PMC5837715

[B35] Haycock P. C. Burgess S. Nounu A. Zheng J. Okoli G. N. Bowden J. (2017). Association between telomere length and risk of cancer and non-neoplastic diseases a mendelian randomization study. *JAMA Oncol.* 3 636–651. 10.1001/jamaoncol.2016.5945 28241208 PMC5638008

[B36] Hernán M. A. Robins J. M. (2006). Instruments for causal inference: an epidemiologist’s dream? *Epidemiology* 17 360–372. 10.1097/01.ede.0000222409.00878.37 16755261

[B37] Hoadley K. A. Yau C. Hinoue T. Wolf D. M. Lazar A. J. Drill E. (2018). Cell-of-origin patterns dominate the molecular classification of 10,000 tumors from 33 Types of cancer. *Cell* 173 291–304.e296. 10.1016/j.cell.2018.03.022 29625048 PMC5957518

[B38] Kachuri L. Saarela O. Bojesen S. E. Davey Smith G. Liu G. Landi M. T. (2018). Mendelian randomization and mediation analysis of leukocyte telomere length and risk of lung and head and neck cancers. *Int. J. Epidemiol.* 48 751–766. 10.1093/ije/dyy140 30059977 PMC6659464

[B39] Khera A. V. Chaffin M. Aragam K. G. Haas M. E. Roselli C. Choi S. H. (2018). Genome-wide polygenic scores for common diseases identify individuals with risk equivalent to monogenic mutations. *Nat. Genet.* 50:1219. 10.1038/s41588-018-0183-z 30104762 PMC6128408

[B40] Khera A. V. Chaffin M. Wade K. H. Zahid S. Brancale J. Xia R. (2019). Polygenic prediction of weight and obesity trajectories from birth to adulthood. *Cell* 177 587–596.e589. 10.1016/j.cell.2019.03.028 31002795 PMC6661115

[B41] Kuo C. L. Pilling L. C. Kuchel G. A. Ferrucci L. Melzer D. (2019). Telomere length and aging-related outcomes in humans: a Mendelian randomization study in 261,000 older participants. *Aging Cell* 18 e13017. 10.1111/acel.13017 31444995 PMC6826144

[B42] Lawlor D. A. Harbord R. M. Sterne J. A. Timpson N. Davey Smith G. (2008). Mendelian randomization: using genes as instruments for making causal inferences in epidemiology. *Statist. Med.* 27 1133–1163. 10.1002/sim.3034 17886233

[B43] Levy D. Neuhausen S. L. Hunt S. C. Kimura M. Hwang S. J. Chen W. (2010). Genome-wide association identifies OBFC1 as a locus involved in human leukocyte telomere biology. *Proc. Natl. Acad. Sci. U.S.A.* 107 9293–9298. 10.1073/pnas.0911494107 20421499 PMC2889047

[B44] Li C. Stoma S. Lotta L. A. Warner S. Albrecht E. Allione A. (2020). Genome-wide association analysis in humans links nucleotide metabolism to leukocyte telomere length. *Am. J. Hum. Genet.* 106 389–404. 10.1016/j.ajhg.2020.02.006 32109421 PMC7058826

[B45] Ma H. X. Zhou Z. Y. Wei S. Liu Z. S. Pooley K. A. Dunning A. M. (2011). Shortened telomere length is associated with increased risk of cancer: a meta-analysis. *PLoS One* 6:e020466. 10.1371/journal.pone.0020466 21695195 PMC3112149

[B46] Machiela M. J. Hofmann J. N. Carreras-Torres R. Brown K. M. Johansson M. Wang Z. (2017). Genetic variants related to longer telomere length are associated with increased risk of renal cell carcinoma. *Eur. Urol.* 72 747–754. 10.1016/j.eururo.2017.07.015 28797570 PMC5641242

[B47] Mangino M. Hwang S. J. Spector T. D. Hunt S. C. Kimura M. Fitzpatrick A. L. (2012). Genome-wide meta-analysis points to CTC1 and ZNF676 as genes regulating telomere homeostasis in humans. *Hum. Mol. Genet.* 21 5385–5394. 10.1093/hmg/dds382 23001564 PMC3510758

[B48] McCarthy S. Das S. Kretzschmar W. Delaneau O. Wood A. R. Teumer A. (2016). A reference panel of 64,976 haplotypes for genotype imputation. *Nat. Genet.* 48 1279–1283. 10.1038/ng.3643 27548312 PMC5388176

[B49] Nelson C. P. Codd V. (2020). Genetic determinants of telomere length and cancer risk. *Curr. Opin. Genet. Dev.* 60 63–68. 10.1016/j.gde.2020.02.007 32171108

[B50] Noyce A. J. Kia D. A. Hemani G. Nicolas A. Price T. R. De Pablo-Fernandez E. (2017). Estimating the causal influence of body mass index on risk of Parkinson disease: a Mendelian randomisation study. *PLoS Med.* 14:e1002314. 10.1371/journal.pmed.1002314 28609445 PMC5469450

[B51] Ojha J. Codd V. Nelson C. P. Samani N. J. Smirnov I. V. Madsen N. R. (2016). Genetic variation associated with longer telomere length increases risk of chronic lymphocytic leukemia. *Cancer Epidemiol. Biomarkers. Prev.* 25 1043–1049. 10.1158/1055-9965.Epi-15-1329 27197291 PMC5008454

[B52] O’Sullivan R. J. Karlseder J. (2010). Telomeres: protecting chromosomes against genome instability. *Nat. Rev. Mol. Cell Biol.* 11 171–181. 10.1038/nrm2848 20125188 PMC2842081

[B53] Pooley K. A. Bojesen S. E. Weischer M. Nielsen S. F. Thompson D. Al Olama A. A. (2013). A genome-wide association scan (GWAS) for mean telomere length within the COGS project: identified loci show little association with hormone-related cancer risk. *Hum. Mol. Genet.* 22 5056–5064. 10.1093/hmg/ddt355 23900074 PMC3836481

[B54] Ripatti S. Tikkanen E. Orho-Melander M. Havulinna A. S. Silander K. Sharma A. (2010). A multilocus genetic risk score for coronary heart disease: case-control and prospective cohort analyses. *Lancet* 376 1393–1400. 10.1016/S0140-6736(10)61267-61266 20971364 PMC2965351

[B55] Rode L. Nordestgaard B. G. Bojesen S. E. (2016). Long telomeres and cancer risk among 95568 individuals from the general population. *Int. J. Epidemiol.* 45 1634–1643. 10.1093/ije/dyw179 27498151

[B56] Shawi M. Autexier C. (2008). Telomerase, senescence and ageing. *Mech. Age. Dev.* 129 3–10. 10.1016/j.mad.2007.11.007 18215413

[B57] Shay J. W. Wright W. E. (2019). Telomeres and telomerase: three decades of progress. *Nat. Rev. Genet.* 20 299–309. 10.1038/s41576-019-0099-91 30760854

[B58] Sheehan N. A. Didelez V. Burton P. R. Tobin M. D. (2008). Mendelian randomisation and causal inference in observational epidemiology. *PLoS Med.* 5:e177. 10.1371/journal.pmed.0050177 18752343 PMC2522255

[B59] Shim H. Chasman D. I. Smith J. D. Mora S. Ridker P. M. Nickerson D. A. (2015). A multivariate genome-wide association analysis of 10 LDL subfractions, and their response to statin treatment, in 1868 Caucasians. *PLoS One* 10:e0120758. 10.1371/journal.pone.0120758 25898129 PMC4405269

[B60] Siegel R. L. Miller K. D. Jemal A. (2019). Cancer statistics, 2019. *CA Cancer J. Clin.* 69 7–34. 10.3322/caac.21551 30620402

[B61] Stewart S. A. Weinberg R. A. (2006). Telomeres: cancer to human aging. *Annu. Rev. Cell Dev. Biol.* 22 531–557. 16824017 10.1146/annurev.cellbio.22.010305.104518

[B62] Sudlow C. Gallacher J. Allen N. Beral V. Burton P. Danesh J. (2015). UK biobank: an open access resource for identifying the causes of a wide range of complex diseases of middle and old age. *PLoS Med.* 12:e1001779. 10.1371/journal.pmed.1001779 25826379 PMC4380465

[B63] Tosto G. Bird T. D. Tsuang D. Bennett D. A. Boeve B. F. Cruchaga C. (2017). Polygenic risk scores in familial Alzheimer disease. *Neurology* 88 1180–1186. 28213371 10.1212/WNL.0000000000003734 PMC5373783

[B64] Verbanck M. Chen C.-Y. Neale B. Do R. (2018). Detection of widespread horizontal pleiotropy in causal relationships inferred from Mendelian randomization between complex traits and diseases. *Nat. Genet.* 50 693–698. 10.1038/s41588-018-0099-97 29686387 PMC6083837

[B65] Went M. Cornish A. J. Law P. J. Kinnersley B. van Duin M. Weinhold N. (2020). Search for multiple myeloma risk factors using Mendelian randomization. *Blood Adv.* 4 2172–2179. 10.1182/bloodadvances.2020001502 32433745 PMC7252541

[B66] Wilson W. R. W. Herbert K. E. Mistry Y. Stevens S. E. Patel H. R. Hastings R. A. (2008). Blood leucocyte telomere DNA content predicts vascular telomere DNA content in humans with and without vascular disease. *Eur. Heart J.* 29 2689–2694. 10.1093/eurheartj/ehn386 18762552

[B67] Wu X. Amos C. I. Zhu Y. Zhao H. Grossman B. H. Shay J. W. (2003). Telomere dysfunction: a potential cancer predisposition factor. *J. Natl. Cancer Inst.* 95 1211–1218. 10.1093/jnci/djg011 12928346

[B68] Xu X. Qu K. Pang Q. Wang Z. Zhou Y. Liu C. (2016). Association between telomere length and survival in cancer patients: a meta-analysis and review of literature. *Front. Med.* 10 191–203. 10.1007/s11684-016-0450-452 27185042

[B69] Yu X. Yuan Z. Lu H. Gao Y. Chen H. Shao Z. (2020). Relationship between birth weight and chronic kidney disease: evidence from systematics review and two-sample Mendelian randomization analysis. *Hum. Mol. Genet.* 29 2261–2274. 10.1093/hmg/ddaa074 32329512

[B70] Yuan J. M. Beckman K. B. Wang R. Bull C. Adams-Haduch J. Huang J. Y. (2018). Leukocyte telomere length in relation to risk of lung adenocarcinoma incidence: findings from the Singapore Chinese Health Study. *Int. J. Cancer* 142 2234–2243. 10.1002/ijc.31251 29318605 PMC5893405

[B71] Zeng P. Wang T. Zheng J. Zhou X. (2019a). Causal association of type 2 diabetes with amyotrophic lateral sclerosis: new evidence from Mendelian randomization using GWAS summary statistics. *BMC Med.* 17:225 10.1186/s12916-019-1448-1449 PMC6892209 31796040

[B72] Zeng P. Yu X. Zhou X. (2019b). Birth weight is not causally associated with adult asthma: results from instrumental variable analyses. *Sci. Rep.* 9:7647 10.1038/s41598-019-44114-44115 PMC6529425 31113992

[B73] Zeng P. Zhou X. (2019a). Causal association between birth weight and adult diseases: evidence from a mendelian randomization analysis. *Front. Genet.* 10:618. 10.3389/fgene.2019.00618 31354785 PMC6635582

[B74] Zeng P. Zhou X. (2019b). Causal effects of blood lipids on amyotrophic lateral sclerosis: a Mendelian randomization study. *Hum. Mol. Genet.* 28 688–697. 10.1093/hmg/ddy384 30445611 PMC6360326

[B75] Zhang C. Doherty J. A. Burgess S. Hung R. J. Lindstrom S. Kraft P. (2015). Genetic determinants of telomere length and risk of common cancers: a Mendelian randomization study. *Hum. Mol. Genet.* 24 5356–5366. 10.1093/hmg/ddv252 26138067 PMC4550826

[B76] Zhang X. Zhao Q. Zhu W. Liu T. Xie S. H. Zhong L. X. (2017). The association of telomere length in peripheral blood cells with cancer risk: a systematic review and meta-analysis of prospective studies. *Cancer Epidemiol. Biomarkers. Prev.* 26 1381–1390. 10.1158/1055-9965.Epi-16-0968 28619828

[B77] Zhu H. Belcher M. van der Harst P. (2011). Healthy aging and disease: role for telomere biology? *Clin. Sci.* 120 427–440. 10.1042/CS20100385 21271986 PMC3035527

